# Sex-specific nutritional and inflammatory trajectories during first-line chemotherapy in older adults with advanced non-small cell lung cancer

**DOI:** 10.3389/fnut.2026.1885061

**Published:** 2026-08-03

**Authors:** Jinxian He, Mingjie Gu, Zhimei Chen, Jiahui Fu, Jian Yu

**Affiliations:** 1 The First Affiliated Hospital with Nanjing Medical University (Jiangsu Province Hospital), Nanjing, China; 2 School of Nursing, Nanjing Medical University, Nanjing, China

**Keywords:** chemotherapy, clinical nutrition, geriatric nutritional risk index, non-small cell lung cancer, older adults, sex differences, supportive care, systemic inflammation response index

## Abstract

**Background:**

Malnutrition and systemic inflammation are closely interrelated determinants of treatment tolerance and prognosis in older adults with advanced non-small cell lung cancer. However, their sex-specific longitudinal patterns during chemotherapy remain insufficiently characterized. This study aimed to describe and compare sex-specific trajectories of nutritional status and systemic inflammation during first-line chemotherapy and to explore their longitudinal association from a clinical nutrition perspective.

**Methods:**

This retrospective longitudinal cohort study included 323 patients aged 60 years or older with stage IIIB–IV non-small cell lung cancer who completed six cycles of first-line chemotherapy. Nutritional status and systemic inflammation were assessed at baseline and before each chemotherapy cycle using the Geriatric Nutritional Risk Index (GNRI) and the Systemic Inflammation Response Index (SIRI), respectively. Sex-specific trajectories were examined using linear mixed-effects models including time, sex, and time-by-sex interaction terms. Sex-stratified multivariable linear regression was then used to evaluate the association between overall changes in GNRI and SIRI after adjustment for tumor stage and histology.

**Results:**

Significant time-by-sex interactions were observed for both GNRI (*P* = 0.019) and SIRI (*P* = 0.002). In male patients, mean GNRI increased progressively across chemotherapy cycles, while mean SIRI decreased over time. Greater reductions in SIRI were independently associated with greater improvements in GNRI (β = −0.224, *P* < 0.001). In female patients, baseline GNRI was higher and remained largely within the no-risk range throughout treatment, whereas SIRI fluctuated without a clear directional trend. No significant association between overall GNRI and SIRI changes was identified in women (*P* = 0.626).

**Conclusion:**

Sex-stratified analyses suggested differential longitudinal relationship between nutritional risk and systemic inflammation during chemotherapy in older adults with advanced non-small cell lung cancer,though formal interaction testing did not confirm statistical significance of this difference. In men, inflammatory monitoring may provide a useful adjunct to nutritional risk assessment. In women, nutritional monitoring may need to focus more on short-term changes in intake, body weight, and treatment-related symptoms than on inflammatory indices alone. These findings support further investigation of sex-informed supportive care strategies in clinical nutrition practice.

## 1 Introduction

Lung cancer remains a leading cause of cancer-related mortality worldwide, and non-small cell lung cancer (NSCLC) accounts for most cases (1). The management of advanced NSCLC in older adults poses a particular clinical challenge. Although chemotherapy remains a cornerstone of treatment, its benefits in this vulnerable population are often limited by increased toxicity and reduced physiological reserve, which can seriously affect treatment tolerance and outcomes (2). Accordingly, optimizing supportive care to manage treatment-related adverse effects and preserve functional status is of particular importance in this population.

Within this context, nutritional status (3, 4) and systemic inflammation (5, 6) have emerged as two closely interrelated determinants of prognosis and potentially modifiable targets in supportive care. Malnutrition is common in cancer patients and frequently forms a vicious cycle with chronic inflammation (7). This interplay contributes to cancer cachexia and functional decline (8), severely impairing patients' quality of life. Therefore, a better understanding of how these processes evolve during treatment may help refine supportive care strategies.

Objective metrics have been developed to quantify these risks. The Geriatric Nutritional Risk Index (GNRI), which integrates serum albumin and body weight, has emerged as a robust prognostic indicator for older cancer patients (9). Similarly, the Systemic Inflammation Response Index (SIRI), derived from routine blood counts, provides a measure of systemic inflammation that independently predicts poorer outcomes in NSCLC (5). However, despite the recognized relationship between nutrition and inflammation (10), little is known about their longitudinal co-evolution during chemotherapy (11). It remains unclear whether changes in one domain precede or drive changes in the other, or if they simply co-vary in response to treatment stress.

Furthermore, the potential moderating role of sex, a fundamental biological variable, in this dynamic is critically underexplored. Well-established sex differences exist in epidemiology, metabolism and body composition. These differences influence cancer risk, treatment response, and side effects (12). For instance, men exhibit a higher incidence of lung cancer and often present with different tumor subtypes compared to women (13). Nevertheless, it remains unclear whether the longitudinal relationship between nutritional and inflammatory changes during chemotherapy differs systematically by sex. Without such evidence, supportive care approaches may overlook clinically relevant differences in vulnerability and recovery patterns between male and female patients.

Given the central role of nutritional risk in treatment tolerance, functional decline, and supportive care decision-making, clarifying whether nutritional status and systemic inflammation evolve differently in male and female patients may have direct relevance for clinical nutrition practice. Therefore, this study aimed (1) to model and compare sex-specific trajectories of GNRI and SIRI across six cycles of first-line chemotherapy in older adults with advanced non-small cell lung cancer, and (2) to examine whether the longitudinal association between changes in these two parameters differed by sex. We hypothesized that male and female patients would exhibit distinct nutrition-inflammation patterns during treatment. By clarifying these differences, this study seeks to inform more individualized nutritional monitoring and supportive care strategies during chemotherapy.

## 2 Materials and methods

### 2.1 Study design and participants

This retrospective longitudinal cohort study was conducted at a tertiary oncology center after approval from the Institutional Review Board of the First Affiliated Hospital of Nanjing Medical University (2024-SR-877). Informed consent was waived by our Institutional Review Board because of the anonymized nature of the data analysis and the retrospective nature of this study. This study was reported in accordance with the Strengthening the Reporting of Observational Studies in Epidemiology (STROBE) statement.

We screened the electronic medical records of patients with NSCLC between January 2022 and June 2025. All included patients received standard first-line platinum-based doublet chemotherapy (carboplatin or cisplatin with pemetrexed or gemcitabine, selected according to histology), administered in routinely scheduled 21-day cycles. According to institutional protocol, cycle delays of up to 7 days were permitted in cases of chemotherapy toxicity. Consequently, the actual inter-cycle intervals across all patients ranged from 21 to 28 days.

**Figure 1:**
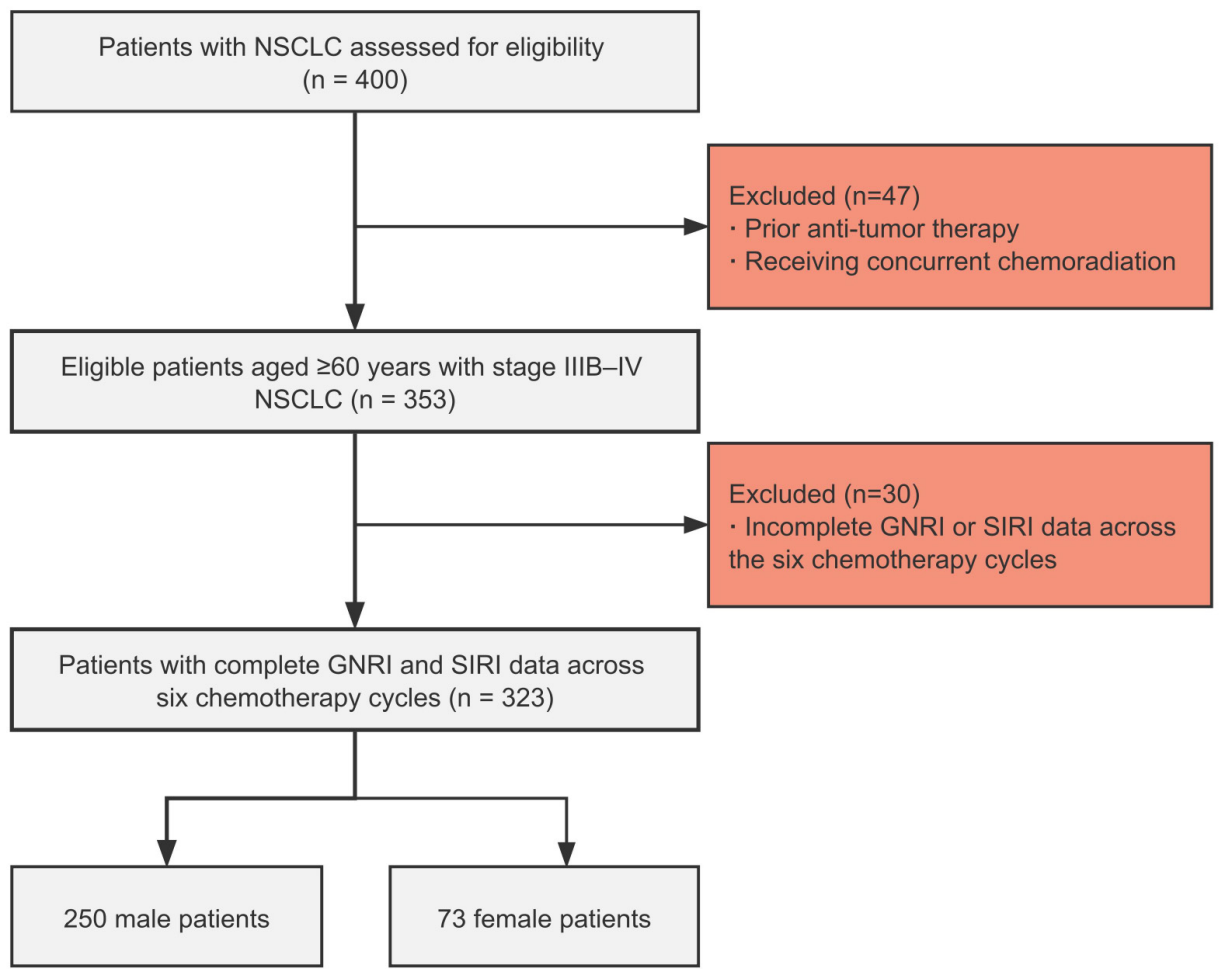
Flow chart of participant selection.

To ensure a homogeneous sample with complete longitudinal data for trajectory modeling, the following inclusion criteria were applied: (1) age ≥ 60 years at diagnosis (defined as older adults in China); (2) cytologically or histologically confirmed NSCLC, clinical stage IIIB-IV according to the 8th edition of the American Joint Committee on Cancer TNM (Tumor, Node, Metastasis) staging system (14); (3) completed at least six cycles of standard first-line chemotherapy; (4) no prior anti-tumor therapy (including surgery, radiotherapy, targeted therapy, or immunotherapy) before the first chemotherapy cycle; and (5) availability of complete data for both nutritional risk and systemic inflammation assessments at all six predefined time points (see below). Exclusion criteria were: (1) a history of other active malignancies (including second primary malignancies), hematological disorders (e.g., leukemia, lymphoma, myelodysplastic syndromes), autoimmune diseases, or chronic infections; (2) documented use of long-term immunosuppressants or corticosteroids (beyond standard premedication) during the study period; and (3) patients receiving concurrent chemoradiation. A total of 400 patients with NSCLC were screened for eligibility, and 323 patients with complete longitudinal data were included in the final analysis (Figure 1). This selection of patients with complete data was critical for the planned sophisticated longitudinal modeling.

### 2.2 Data collection and measurements

Clinical and laboratory data were extracted from electronic medical records at six predefined and clinically consistent timepoints: T1 (baseline, 24–48 hours before the first chemotherapy cycle) and T2-T6 (24–48 hours before the second to sixth cycles, respectively). These repeated assessments were designed to capture longitudinal changes in nutritional risk and systemic inflammation during treatment.

#### 2.2.1 Geriatric nutritional risk index (GNRI)

The Geriatric Nutritional Risk Index (GNRI) was selected for this study because it is a validated, objective, and easily calculable tool that integrates serum albumin and body weight, both of which are routinely measured in clinical practice. Unlike subjective tools GNRI does not require additional patient interviews and is therefore well-suited for retrospective analysis. The Geriatric Nutritional Risk Index (GNRI) was calculated according to the validated formula proposed by Bouillanne et al. (9):


$$\begin{array}{l} \text{G}\text{N}\text{R}\text{I}=\left[1.489\;\times \text{s}\text{e}\text{r}\text{u}\text{m}\;\text{a}\text{l}\text{b}\text{u}\text{m}\text{i}\text{n}\left(\text{g}/\text{L}\right)\right]\\ \;+\left[41.7\times \left(\frac{\text{actual body weight }}{\text{ideal body weight}}\;\right)\right]. \end{array}$$

Ideal body weight was determined by the Lorentz formula, stratified by sex (15):

For males: ideal body weight (kg) = Height (cm)−100–[(*Height*− 150)/4].

For females: ideal body weight (kg) = Height (cm) −100–[(*Height* − 150)/2.5].

If actual weight exceeded the ideal weight, the ratio was set to 1 to avoid overestimating nutritional status. Nutrition risk was categorized into 4 grades according to GNRI values: high risk (GNRI: < 82), moderate risk (GNRI: 82 to < 92), low risk (GNRI: 92 to ≤ 98), and no risk (GNRI: >98). GNRI is a validated index for assessing nutritional risk and predicting clinical outcomes in older adults and patients with advanced NSCLC (16, 17).

#### 2.2.2 Systemic inflammationResponse index (SIRI)

The Systemic Inflammation Response Index (SIRI) is a cost-effective hematologic biomarker derived from peripheral blood cell counts, commonly used to evaluate systemic inflammation status in clinical research (5). Its calculation integrates neutrophil, monocyte, and lymphocyte counts, and it has been validated for prognostic predictions in cancer (18, 19). Higher SIRI values indicate stronger systemic inflammation and are associated with poorer clinical outcomes in cancer.

SIRI is calculated using the following equation:


$$\begin{array}{l} \text{S}\text{I}\text{R}\text{I}\;=\;\left(\frac{\text{N}\text{e}\text{u}\text{t}\text{r}\text{o}\text{p}\text{h}\text{i}\text{l}\;\text{C}\text{o}\text{u}\text{n}\text{t}\;\times \;\text{M}\text{o}\text{n}\text{o}\text{c}\text{y}\text{t}\text{e}\;\text{C}\text{o}\text{u}\text{n}\text{t}}{\text{L}\text{y}\text{m}\text{p}\text{h}\text{o}\text{c}\text{y}\text{t}\text{e}\;\text{C}\text{o}\text{u}\text{n}\text{t}}\;\right). \end{array}$$

#### 2.2.3 Covariates

Baseline demographic and clinical variables were collected, including age, sex, tumor stage (AJCC 8th edition), smoking status (never, former, and current), drinking status (never, former, and current), and Eastern Cooperative Oncology Group Performance Status (ECOG-PS) (20).

### 2.3 Statistical analysis

Continuous variables were summarized as mean±standard deviation (SD) and compared using independent-samples *t*-tests. Categorical variables were expressed as frequencies (%) and compared using the chi-square test or Fisher's exact test, as appropriate. Sex-specific longitudinal trajectories of GNRI and SIRI were analyzed using linear mixed-effects models with time, sex, and a time-by-sex interaction term as fixed effects. To further quantify the overall longitudinal association between nutritional and inflammatory changes, for each patient, change scores (Δ) were calculated as the difference between the value at each subsequent timepoint (T2 to T6) and the baseline value at T1 (i.e., ΔGNRI_*t* = GNRI_*t* – GNRI_T1, and same for SIRI), and patient-level mean change values (mean ΔGNRI and mean ΔSIRI) were derived. Sex-stratified multivariable linear regression models were then fitted with mean ΔGNRI as the dependent variable and mean ΔSIRI as the main independent variable, with adjustment for tumor stage and histology. All tests were two-sided, and *P* < 0.05 was considered statistically significant. Analyses were conducted using R software (version 4.4.3; R Foundation for Statistical Computing, Vienna, Austria).

## 3 Results

### 3.1 Baseline patient characteristics

A total of 323 patients were included, comprising 250 males (77.4%) and 73 females (22.6%). As summarized in Table 1, no significant difference in age was observed between sexes (70.37 ± 5.99 vs. 68.90 ± 5.88 years, *P* = 0.064). Significant sex disparities were noted in histology distribution (*P* < 0.001), with squamous cell carcinoma being more prevalent in males (128/250, 51.2%) and adenocarcinoma in females (59/73, 80.8%). Tumor stage distribution also differed significantly (*P* < 0.001), with a higher proportion of stage IV disease in females (86.3% vs. 50.4% in males). Smoking and drinking histories were strongly associated with sex (both *P* < 0.001), with most females being never-smokers and never-drinkers.

**Table 1:** Baseline characteristics of older adults with NSCLC (*n* = 323).

Characteristic	Male (*n* = 250)	Female (*n* = 73)	*P*
**Age, years (mean** **±SD)**	70.37 ± 5.99	68.90 ± 5.88	0.064
Histology, *n* (%)			
Squamous cell carcinoma	128	9	< 0.001
Adenocarcinoma	107	59	
Adenosquamous carcinoma	15	5	
Tumor stage, *n* (%)			
IIIB	124	10	< 0.001
IV	126	63	
ECOG-PS,*n* (%)			
0	10	7	0.132
1	152	45	
2	88	21	
Smoking status, *n* (%)			
Never	32	67	< 0.001
Former	144	3	
Current	74	3	
Drinking status, *n* (%)			
Never	119	68	< 0.001
Former	10	1	
Current	121	4	
**Baseline GNRI (mean** **±SD)**	94.93 ±6.90	98.18 ± 6.08	< 0.001
**Baseline SIRI (mean** **±SD)**	2.48 ± 1.41	2.06 ± 0.98	0.179

*NSCLC*, non-small cell lung cancer; *ECOG-PS*, eastern cooperative oncology group performance status; *GNRI*, geriatric nutritional risk index; *SIRI*, systemic inflammation response index; *SD*, standard deviation.

Notably, baseline GNRI was significantly lower in males than in females (94.93 ± 6.90 vs. 98.18 ± 6.08, *P* < 0.001), placing the average male patient in the “low risk” category and the average female patient in the “no risk” category. Baseline SIRI did not differ significantly between groups (2.48 ± 1.41 vs. 2.06 ± 0.98, *P* = 0.179).

### 3.2 Sex-Specific longitudinal trajectories of GNRI and SIRI

To evaluate whether nutritional risk and systemic inflammation evolved differently by sex during chemotherapy, linear mixed-effects models were fitted for GNRI and SIRI across the six treatment cycles. After adjustment for tumor histology and stage, significant time-by-sex interaction effects were identified for both GNRI (*F* = 5.593, *P* = 0.019) and SIRI (*F* = 9.357, *P* = 0.002), indicating distinct longitudinal patterns in male and female patients.

**Figure 2:**
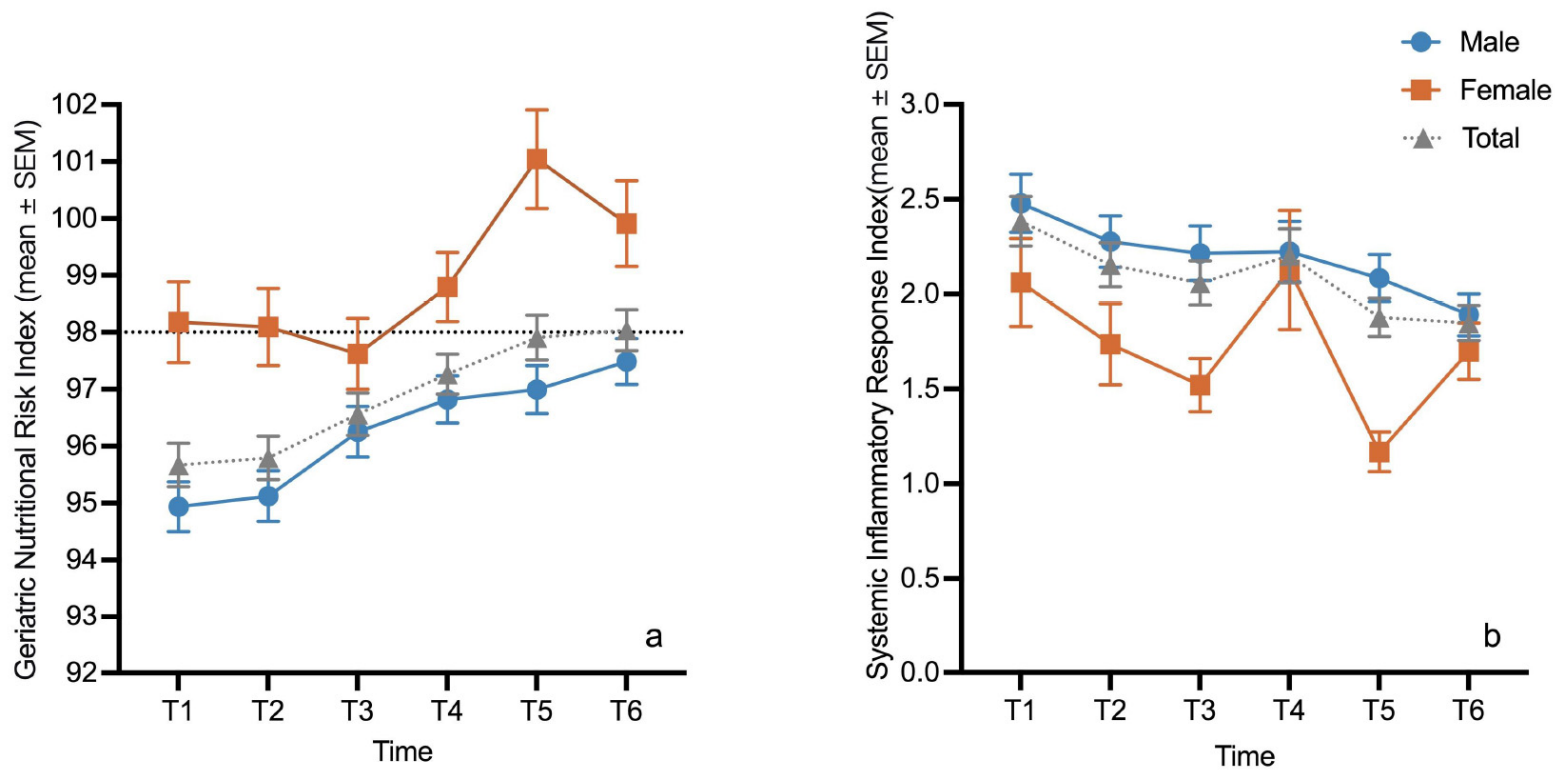
Sex-specific longitudinal trajectories of nutritional status and systemic inflammation. **(a)** Changes in GNRI over six chemotherapy cycles. The dashed horizontal line indicates the threshold between low risk and no risk (GNRI = 98). **(b)** Changes in SIRI over six chemotherapy cycles. In both panels, blue lines represent male patients, red lines represent female patients, and gray dashed lines represent the overall cohort trajectory (both sexes combined) for reference. *SEM*, standard error of the mean.

As illustrated in Figure 2, male patients showed a general increase in GNRI (from 94.9 at T1 to 97.5 at T6) alongside a decrease in SIRI (from 2.36 to 1.79). In contrast, female patients, while maintaining a mean GNRI above the “no-risk” threshold (> 98), did not exhibit a statistically significant linear trend over time. Their SIRI trajectory also varied over time without showing a clear directional pattern. For reference, the overall cohort trajectory (both sexes combined) is also shown.

### 3.3 Dynamic association between changes in GNRI and SIRI

To quantify the relationship between the overall inflammatory and nutritional changes throughout chemotherapy, multiple linear regression analysis was performed using the patient-level mean change scores (mean ΔSIRI and mean ΔGNRI). After adjustment for tumor stage and histology, mean ΔSIRI was a significant independent predictor of mean ΔGNRI in male patients (β = −0.224, 95% CI: −0.248 to −0.072, *P* < 0.001). This association was not observed in female patients (β = −0.059, 95% CI: −0.200 to 0.121, *P* = 0.626). The detailed results of these stratified regression analyses are presented in Table 2.

**Table 2:** Multiple linear regression analysis for the association between overall trends of nutritional and inflammatory changes, stratified by sex.

Parameter	B	SE	Beta	*t*	*P*	Lower 95%CI	Upper 95 CI
Male patients(*n* = 250)							
Constant	0.806	0.324		2.485	0.014	0.167	1.445
mean ΔSIRI	−0.160	0.045	−0.224	−3.586	< 0.001	−0.248	−0.072
Tumor stage (IIIB = 1, IV = 2)	−0.235	0.18	−0.085	−1.308	0.192	−0.59	0.119
Adenocarcinoma = 1	Ref.						
Squamous cell carcinoma = 2	0.032	0.185	0.012	0.173	0.862	−0.333	0.398
Adenosquamous Carcinoma = 3	0.041	0.377	0.007	0.109	0.913	−0.702	0.784
Female patients (*n* = 73)							
Constant	−0.786	0.963		−0.816	0.417	−2.707	1.135
mean ΔSIRI	−0.039	0.08	−0.059	−0.49	0.626	−0.2	0.121
Tumor stage (IIIB = 1, IV = 2)	0.525	0.497	0.133	1.055	0.295	−0.468	1.517
Adenocarcinoma = 1	Ref.						
Squamous Cell carcinoma = 2	0.746	0.522	0.181	1.43	0.157	−0.295	1.787
Adenosquamous carcinoma = 3	0.688	0.639	0.128	1.077	0.285	−0.587	1.964

*SE*, standard error; *GNRI*, geriatric nutritional risk index; *SIRI*, systemic inflammation response index

## 4 Discussion

In this retrospective longitudinal cohort of older adults with advanced non-small cell lung cancer receiving first-line chemotherapy, we observed clear sex-specific differences in the evolution of nutritional risk and systemic inflammation. From a clinical nutrition perspective, the principal finding is that improvements in nutritional status among men occurred in parallel with reductions in inflammatory burden, whereas in women, nutritional status remained comparatively preserved and was not significantly associated with inflammatory change.

In male patients, improvements in GNRI over time coincided with reductions in SIRI, and overall changes were significantly correlated. This coordinated pattern suggests that, in men, nutritional status and systemic inflammation may reflect a shared underlying physiological process during chemotherapy. One possible explanation is that, by controlling tumor burden or disease-related symptoms, chemotherapy may be associated with reduced inflammation and cancer-related catabolism, thereby creating conditions more favorable for nutritional recovery (21–24). Because albumin is a negative acute-phase reactant and a major component of the GNRI formula, while reflecting nutritional status, it may also partially reflect systemic inflammation. Consistent with this biological interplay, the parallel improvement in GNRI and reduction in SIRI observed in male patients during chemotherapy is clinically reasonable, as tumor burden and systemic inflammation decrease, both nutritional recovery and inflammation-mediated normalization of serum albumin may occur concurrently. Therefore, future studies that include synchronous measurement of CRP or interleukin-6 with albumin at each time point are needed to further clarify the degree to which inflammation-related changes in albumin contribute to the observed GNRI trajectories. Additionally, supportive care measures such as antiemetic therapy, dietary counseling, and nutritional supplements probably contribute to maintaining caloric intake and preserving muscle mass, supporting the observed improvement in nutritional risk among male patients (25, 26).

In contrast, female patients maintained GNRI values predominantly within the no-risk range throughout treatment, whereas their inflammatory markers showed greater variability and no significant association with changes in GNRI. This pattern may suggest that nutritional status in women was relatively less tightly coupled with systemic inflammation in the present cohort. Potential explanations, though speculative and not directly tested in this study, may include sex-related differences in body composition, metabolic reserve, hormonal regulation, or symptom profiles during chemotherapy (27–29). It is also important to note that the smaller female sample size may have limited the ability to detect subtler associations in this subgroup.

While the point estimates differ between sexes in stratified analyses, we cannot definitively conclude that the strength of the nutritional-inflammatory association is statistically distinct across male and female patients at the conventional significance level. The observed pattern may reflect genuine but modest biological sex differences in the coupling between systemic inflammation and nutritional status during chemotherapy, with the interaction term underpowered to detect statistical significance given the smaller female subgroup. Consequently, our findings should be interpreted as hypothesis-generating rather than confirmatory, and warrant replication in adequately powered prospective studies designed specifically to test sex-by-inflammation interactions in nutritional trajectories.

The observed sex differences have practical implications for supportive care. In male patients, the coordinated improvement in GNRI and reduction in SIRI suggests that inflammatory monitoring may complement routine nutritional assessment during chemotherapy. When inflammatory indices remain elevated or increase over time, clinicians may consider earlier nutritional reassessment, including review of body weight, serum albumin, oral intake, and treatment-related symptoms (30). In female patients, inflammatory indices alone may be less informative for identifying emerging nutritional vulnerability. Instead, closer attention to short-term changes in dietary intake, nausea, vomiting, fatigue, and body weight may be more clinically useful for timely nutritional intervention (31).

The strengths of this study include its longitudinal design and its focus on sex as an often overlooked variable. Several limitations should be considered. First, the retrospective single-center design and the inclusion requirement of six completed chemotherapy cycles may have introduced selection bias toward patients with better treatment tolerance and a more favorable clinical course, thereby limiting generalizability to the broader advanced NSCLC population. Patients who discontinued treatment due to disease progression, severe toxicity, or death were excluded, which may result in an overestimation of the observed improvements in nutritional status and inflammatory indices, as these excluded patients would likely have exhibited deteriorating trajectories. Second, although GNRI is a validated nutritional risk index, it does not directly capture dietary intake, skeletal muscle mass, fat mass, or cachexia-related body composition changes. Third, direct information on nutritional interventions during chemotherapy, such as oral nutritional supplements, or albumin therapy, or individualized dietary counseling, was not available, which limits interpretation of the mechanisms underlying GNRI improvement and precludes attribution of the observed changes specifically to chemotherapy-related effects. Fourth, although we excluded patients with documented active malignancies, autoimmune diseases, or chronic infections, we did not systematically collect a validated comorbidity index for all patients, and other unmeasured comorbidities may have contributed to residual confounding. Future prospective studies should incorporate a validated comorbidity index to adjust for these factors. Finally, the smaller female sample size may have reduced statistical power to detect more subtle associations in women. Prospective multicenter studies incorporating dietary intake, body composition, and treatment toxicity are needed.

## 5 Conclusion

This study indicates that male and female patients with advanced non-small cell lung cancer may exhibit different patterns in the longitudinal relationship between nutritional risk and systemic inflammation during first-line chemotherapy. From a clinical nutrition perspective, rising inflammatory indices may serve as an adjunctive warning signal for nutritional deterioration in older men, whereas nutritional monitoring in older women may need to rely more on short-interval assessment of intake, body weight, and treatment-related symptoms. Overall, these findings support the development of more individualized, sex-informed supportive care strategies in oncology nutrition, and highlight the need for prospective validation in larger cohorts.

## Data Availability

The raw data supporting the conclusions of this article will be made available by the authors, without undue reservation.
